# Evaluation of the Double Agar Gel Immunodiffusion Test and of the Enzyme-Linked Immunosorbent Assay in the Diagnosis and Follow-Up of Patients with Chronic Pulmonary Aspergillosis

**DOI:** 10.1371/journal.pone.0134841

**Published:** 2015-08-13

**Authors:** Priscila Zacarias de Azevedo, Tatiane Fernanda Sylvestre, Ricardo de Souza Cavalcante, Lídia Raquel de Carvalho, Daniela Vanessa Moris, Maria Luiza Cotrim Sartor de Oliveira, Rinaldo Poncio Mendes

**Affiliations:** 1 Tropical Diseases Department, Faculdade de Medicina de Botucatu, Universidade Estadual Paulista (UNESP), Botucatu, São Paulo State, Brazil; 2 Instituto de Biociências de Botucatu, UNESP, Botucatu, São Paulo State, Brazil; 3 Universidade do Oeste Paulista, UNOESTE, Presidente Prudente, São Paulo State, Brazil; 4 Department of Pathology, Faculdade de Medicina de Botucatu, Universidade Estadual Paulista (UNESP), Botucatu, São Paulo State, Brazil; Hans-Knoell-Institute (HKI), GERMANY

## Abstract

The diagnosis of chronic pulmonary aspergillosis (CPA) depends on the radiologic image and the identification of specific antibodies. The present study aimed to evaluate accuracy parameters of enzyme-linked immunosorbent assay (ELISA) and of the determination of serum galactomannan level in the diagnosis of patients with CPA, comparing these results with the double agar gel immunodiffusion (DID) test. In addition, the prevalence of cross-reactivity and the serological progression after treatment were evaluated by comparing DID and ELISA. Six study groups were formed: G1: 22 patients with CPA, 17 of whom had *Aspergillus* fungus ball, one chronic cavitary pulmonary aspergillosis (CCPA) and four chronic fibrosing pulmonary aspergillosis (CFPA); G2: 28 patients with pulmonary tuberculosis (TB); G3: 23 patients with histoplasmosis (HST); G4: 50 patients with paracoccidioidomycosis (PCM); G5: 20 patients with cryptococcosis (CRC); and G6: 200 healthy controls. Serum antibodies were measured by DID and ELISA, with two antigen preparations—*Aspergillus fumigatus* (DID_1_, ELISA_1_) and a pool of *A*. *fumigatus*, *A*. *flavus* and *A*. *niger* antigens (DID_2_, ELISA_2_). The Platélia *Aspergillus* Enzyme Immunoassay (EIA) kit was used to measure galactomannan. The *cut-off* points of ELISA were determined for each antigen preparation and for the 95% and 99% confidence intervals. Despite the low sensitivity, DID was the technique of choice due to its specificity, positive and negative predictive values and positive likelihood ratio–especially with the antigen pool and due to the low frequency of cross-reactivity. ELISA_1_ and a 0.090 *cut-off* showed high sensitivity, specificity and negative predictive value, but a high frequency of cross-reactivity with CRC. The best degree of agreement was observed between ELISA_1_ and ELISA_2_. The detection of serum galactomannan showed high sensitivity, comparable to ELISA_2_. The immunodiffusion test showed an excellent relationship with the progression after treatment, which made it the reaction of choice for patient follow-up.

## Introduction

The genus *Aspergillus* contains approximately 150 confirmed species, and others continue to be described [1]. However, only a few species cause human disease, with evident predominance of *A*. *fumigatus*, *A*. *flavus*, *A*. *niger*, *A*. *terreus* and *A*. *nidulans* [1]. *A*. *fumigatus* is the most common etiologic agent of invasive and non-invasive aspergillosis, including cases of pulmonary disease [1, 2].

The host-parasite interaction between *Aspergillus* spp. and humans is highly diverse. In patients with neutropenia, this interaction presents as invasive pulmonary aspergillosis; in some hyperergic patients, as allergic bronchopulmonary aspergillosis; in patients with no obvious cause of immunosuppression, as CPA. The latter has been frequently observed in patients with pulmonary sequelae, such as those observed in pulmonary TB [3] and in chronic obstructive pulmonary disease [4]. In areas where HST is hyperendemic, CPA is widely reported [5]. AIDS patients, whose immune deficiency is linked to the destruction of CD4^+^ T lymphocytes, are less affected by *Aspergillus* spp. [6].

Aspergillosis presents in various clinical forms, among them chronic pulmonary aspergillosis (CPA), which in turn is divided into aspergilloma, chronic cavitary pulmonary aspergillosis (CCPA) and chronic fibrosing pulmonary aspergillosis (CFPA) [3]. Aspergilloma, also called “*Aspergillus* fungus ball” (aspergilloma), is the most frequent form of CPA and generally affects patients with tuberculous lung cavity [7].

The identification of specific serum antibodies, determined by double agar gel immunodiffusion (DID) test [8, 9], is important for the diagnosis of pulmonary aspergillosis. Few studies have evaluated the use of the enzyme immunoassays and the determination of serum galactomannan (GM) in the diagnosis of different forms of CPA [10–12].

Surgical intervention with resection of one or more lung segments was the treatment of choice for cases of aspergilloma [13]. However, the surgery is followed by a mortality rate that ranges from 7% to 23% [14–17]. Itraconazole, a triazole with good diffusion into the lung cavity colonized by *Aspergillus* spp., is effective for the treatment of aspergilloma [13, 18–20]. The treatment, which is maintained for a long time, can be controlled by clinical, radiological and serological evaluation, especially when antifungal agents such as itraconazole are used. However, few studies have evaluated the serological progression of patients with aspergilloma under antifungal treatment [16–19].

The present study aimed to evaluate the accuracy of enzyme-linked immunosorbent assay (ELISA) and of serum GM level in the diagnosis of patients with CPA and to compare them with DID. In addition, the serological follow-up of these patients was evaluated with the introduction of an antifungal agent, comparing ELISA with DID.

## Patients and Methods

A complex, retrospective and prospective study was performed with 25 patients with CPA who were treated at the Tropical Diseases Ward and at the South American Blastomycosis (Paracoccidioidomycosis) Clinic of the School of Medicine of Botucatu—São Paulo State University (Universidade Estadual Paulista—UNESP), where patients with other systemic mycoses are also treated.

### Study population

#### Patients, case definition and inclusion and exclusion criteria

Patients with CPA, tuberculosis (TBC), histoplasmosis (HST), cryptococcosis (CRC) and paracoccidioidomycosis (PCM) were studied.

#### Patients with CPA

Patients with CPA (G1) in different clinical forms were evaluated: aspergilloma, CCPA and CFPA. Based on specifications by Denning et al. [3, 21] and Camuset et al. [22], the case definitions used in the present study are presented below.

#### Aspergilloma

Cases of aspergilloma exhibited a clinical picture and radiography and/or planigraphy and/or chest computed tomography (CT) consistent with lung cavitation and solid rounded mass inside it, which is suggestive of fungus ball.

#### CCPA

Cases of CCPA exhibited a clinical picture and radiography and/or planigraphy and/or chest CT scan consistent with lung cavitation and no solid rounded mass inside it.

#### CFPA

Cases of CFPA exhibited clinical picture, and radiography, and/or planigraphy and/or chest CT scan consistent with pulmonary fibrosis and no cavitation.

All aspergilloma, CCPA and CFPA cases were classified as confirmed, probable and possible cases, as follows:

#### Confirmed case

Characterized by the presence of specific serum antibodies determined by the DID test or by the identification of *Aspergillus* sp. in bronchoalveolar lavage.

#### Probable case

Characterized by the presence of hyaline hyphae consistent with *Aspergillus* sp. in the sputum.

#### Possible case

Characterized by negative results on specific anti-*Aspergillus* serum antibody detection by DID test, as well as the absence of hyaline hyphae consistent with *Aspergillus* sp. in the sputum.

The inclusion and exclusion criteria were the same for the different clinical forms of CPA.

#### Inclusion criteria

Confirmed and probable aspergilloma, CCPA and CFPA patients were included in the study.

#### Exclusion criteria

Patients with AIDS or extra-pulmonary lesion caused by systemic diseases of inflammatory or neoplastic origin as comorbidity, pregnancy and lactation were excluded from the study.

### Patients with pulmonary tuberculosis

In total, 28 patients who had pulmonary TB confirmed (G2) by the identification of acid-fast bacilli (AFB) in the sputum, in a histopathological examination of a lung fragment stained by the Ziehl-Neelsen method and/or in a culture of these clinical samples in Lowenstein-Jensen medium were included in the study. These patients presented no underlying diseases and responded to the classic treatment regimen for *Mycobacterium tuberculosis*.

### Patients with histoplasmosis, paracoccidioidomycosis and cryptococcosis

In total, 23 patients with histoplasmosis—HST (G3), 50 with paracoccidioidomycosis—PCM (G4) and 20 with cryptococcosis—CRC (G5) presenting clinical condition consistent with each fungal disease and direct mycological and / or histopathological examination of clinical samples (confirmed cases), or without the identification of the fungus but with positive detection of specific serum antibodies by DID test (HST and PCM), or of the specific antigen by the latex agglutination test (probable cases) were included in the study [23].

#### Exclusion criteria

The exclusion criteria were the same for patients with pulmonary TBC and PCM. Patients with other infectious, inflammatory or neoplastic systemic diseases as comorbidities, pregnancy and lactation. Patients with AIDS were excluded from the study. The exclusion criteria for patients with HST and CRC were AIDS, pregnancy and lactation.

### Healthy individuals

The control group consisted of 200 healthy individuals (G6) who were blood donors of the Blood Center of Botucatu.

### Study design

The study was designed to compare the accuracy parameters of two methods in the detection of serum antibodies (DID and ELISA) using two types of antigen preparations (*A*. *fumigatus* antigen and *Aspergillus* spp. antigen pool) and serum measurement of GM. The rate of cross-reactivity with sera from patients with other infectious diseases that are part of the differential diagnosis for CPA was also determined. All evaluations were performed with sera collected when the patients were admitted.

In addition, the serological follow-up of patients with CPA under treatment was evaluated by comparing two serological methods (DID and ELISA) and two antigen preparations (*A*. *fumigatus* antigen and *Aspergillus* spp. antigen pool). Serum samples obtained before the treatment started and at three time points after it started were used in the study.

### Score of symptoms and signs

Symptoms and signs were periodically evaluated. Every complaint was scored by the same physician (RSC) on a single 0–4 system, based on De Beule et al. (1988) specifications, with some modifications: 0- absent, 1-mild, 2-moderate, 3- intense, and 4-very intense. Cough, expectoration, dyspnea, and thoracic pain were scored using this schedule. The sputum was scored as without blood (score 0), blood-streaked sputum (score 1), gross blood sputum (score 2), a coffee cup of blood—up to 50 mL a day (score 3), and more than 50 mL of blood a day (score 4). Weight-loss was classified in relationship to the usual body weight as absent / score 0, mild / score 1 (<5%), moderate / score 2 (5–10%), intense /score 3 (between 11 and 20%), and very intense (>20%). Fever was scored in relationship to the axillary temperature: absent / score 0 (<37,0°C), mild / score 1 (37°–38°C), moderate / score 2 (38°C–39°C), intense / score 3 (39°C–40°C or >38°C more than 3 times per week), and very intense score 4 (>40°C or >39°C daily). The global score was the sum of the scores given to every complaint. The scores given at admission served as a baseline with which the subsequent evaluations were compared. A careful evaluation of the scores was performed to standardize the inter-patients analysis [24].

## Methods

### Antigens used

The following antigens, obtained by culture filtrate, were used: a) *A*. *fumigatus* (DID), produced at the Laboratory of Clinical Mycology of the School of Pharmaceutical Sciences of Araraquara–UNESP; b) *A*. *fumigatus* (DID_1_), produced at the Laboratory of Immunodiagnosis of Mycoses—Adolfo Lutz Institute of São Paulo (Instituto Adolfo Lutz de São Paulo), São Paulo; c) antigen pool—*A*. *fumigatus*, *A*. *flavus* and *A*. *niger* (DID_2_), produced at the Laboratory of Clinical Mycology of the School of Pharmaceutical Sciences of Araraquara–UNESP.

This study was carried out with serum samples from our serum bank at -80°C. DID was the immunodiffusion test performed with *A*. *fumigatus* antigen when each patient was clinically evaluated. Thus, different antigen batches were used along the years. When this study was performed, we used two different antigen preparations recently prepared; all the reactions were carried out with the same batch antigen. The comparison between the DID and DID_1_ showed difference of intensity no higher than one dilution.

### Determination of serum antibodies and GM level

#### Agar gel DID

The serum levels of anti-*Aspergillus* antibodies (*A*. *fumigatus* and antigen pool) were determined by DID, according to the specifications of Restrepo et al. [8, 9], at the Tropical Diseases Research Laboratory of the School of Medicine of Botucatu–UNESP. The tests were performed upon the admission of each patient (DID) and when the present study was conducted (DID_1_ and DID_2_).

The tests were performed with undiluted serum followed by two-fold dilutions starting with 1:2. For each test, a positive and a negative control serum were included.

#### Detection of antibodies by the enzyme immunoassay (ELISA)–indirect method

The serum levels of anti-*Aspergillus* antibodies were determined by ELISA [25, 26], according to the standard protocol of the Tropical Diseases Research Laboratory of the School of Medicine of Botucatu–UNESP. All tests were performed at the same place, and the samples were processed in duplicate.

The plates (NUNC—MaxiSorp) were pre-sensitized with 10 μL of *A*. *fumigatus* antigen and *Aspergillus* spp. antigen pool (*A*. *fumigatus*, *A*. *flavus* and *A*. *niger*) and incubated for 2 hours at 37°C and then for 18 hours in the refrigerator. Subsequently, five washes were performed with 300 μL of phosphate-buffered saline with 0.1% Tween (0.1% PBS-T: blocking buffer) in a plate washer (Biotek-Elx 50). The wells were then filled with 100 μL of PBS-T 0,1% with bovine serum albumin (BSA- Sigma), and maintained at room temperature for 1 hour. Next, the serum samples were diluted to 1:100, and 100 μL were added to the microplate and incubated for 1 hour at 37°C. Then, five washes were performed with 300 μL of the blocking buffer solution and goat anti-human immunoglobulin G (IgG) marked with peroxidase (Sigma) diluted to 1:3000; next, the plates were incubated for 1 hour at 37°C. Subsequently, five washes were performed with 300 μL of the sodium phosphate buffer solution with 0.1% Tween and 0.1% PBS-T, and the developing solution was prepared with 200 μL of tetramethylbenzidine (TMB) and 2 μL of 30% H_2_O_2_ in 10 mL of citrate-acetate buffer at pH 6.0. A volume of 100 μL was added to each well, and the reaction was allowed to proceed for 30 minutes at 37°C in an oven. The reaction was interrupted with 50 μL of 4 N sulfuric acid solution. The spectrophotometric reading (Bio-Rad, Mark Microplate Reader) was performed at 450 nm. For each test, a positive and a negative serum were included.

#### GM detection by sandwich ELISA

Serum samples stored at -80°C were processed for sandwich ELISA (Bio-Rad Platélia *Aspergillus* EIA—62796) according to the manufacturer’s instructions.

#### Serological follow-up of patients treated with itraconazole

The serological follow-up of 10 patients with CPA was evaluated using ELISA and DID. The clinical and serological evaluations were performed before the treatment started, which was defined as the 0 time (T_0_), and periodically until the patients were clinically cured, called time 3 (T_3_). Clinical cure was defined as the disappearance of the symptoms previously exhibited by the patient. Between these two times, the patient was evaluated at two other times, which were defined in relation to the number of weeks of treatment: T_1_: 4 to 6; T_2_: 7 to 10.

#### Determination of the *cut-off* point of the ELISA test

The *cut-off* point of the ELISA test was defined by the construction of the receiver operator characteristic (ROC) curve, using confidence intervals of 95% and 99%, according to the specifications of Fletcher & Fletcher [27] and Frei et al. [28].

### Accuracy parameters

The accuracy parameters were determined in 22 patients with CPA and in 200 healthy individuals (controls) to determine the sensitivity, specificity, positive predictive value (PPV), negative predictive value (NPV), accuracy (Ac), positive likelihood ratio (PLR) and negative likelihood ratio (NLR), also called verisimilitude ratio, according to the specifications of Fletcher & Fletcher [27].

When calculating the likelihood ratios, when one of the frequencies was zero, this value was replaced by 1.0 and the likelihood ratio was called corrected positive or negative likelihood ratio (CPLR or CNLR) [29].

### Cross-reactivity with sera from patients with other granulomatous diseases

The sera of 20 patients with CRC, 23 patients with HST, 28 patients with TB and 50 patients with PCM were used to test the cross-reactivity ratio with diseases that can be considered part of the differential diagnosis of CPA.

### Ethics statement

The project was approved by the Institutional Ethics Research Committee (Process no. 210.781-CEP). The informed consent form was signed by the patients included in the prospective study.

### Statistical analysis of results

The likelihoods in dependent populations were compared by Cochran’s Q test, according to the specifications of Curi [30], followed by McNemar’s test, according to Siegel [31]. The degree of agreement between both the two tests was evaluated using the kappa coefficient, according to the specifications of Landis et al. [32]. The kappa coefficient was interpreted as follows: (a) poor: when below 0.00; (b) slight: between 0.00 and 0.20; (c) fair: between 0.21 and 0.40; (d) moderate: between 0.41 and 0.60; (e) substancial: between 0.61 and 0.80; and (d) almost perfect: between 0.81 and 1.00 [32]. For each statistical test, the differences were considered significant when *p* ≤ 0.05.

## Results

The survey of the 25 patients with CPA showed the presence of 20 cases of aspergilloma (confirmed—16; probable—1; possible—3), one with CCPA (probable) and four CFPA (confirmed—3; probable—1). The predominant clinical manifestations were cough (77.3%), weight loss (72.7%) and hemoptysis (63.6%). The CPA data were analysed according to the procedures performed, time between the underlying disease and CPA, concomitant diseases and use of antifungal compounds. In the present study, confirmed and probable cases were included, totalling 22 patients.

The procedure most frequently performed in these patients was transbronchial biopsy. The identification of *Aspergillus* spp. and pulmonary fibrosis were the most common histopathological findings. The time between the underlying disease and the diagnosis of CPA was highly variable, with a median of 5 years (range, 0–45 years). TB was the most prevalent underlying disease. Itraconazole was the antifungal compound used by most patients. Finally, there was a predominance of satisfactory responses to treatment (Table 1).

**Table 1:** Characterization of the 25 evaluated patients with chronic pulmonary aspergillosis.

**Age**	Median = 55 (33–80)			
**Sex**	Male = 20 (80%)		Female = 5 (20%)	
**Underlying diseases**	TB = 19 (76%)	PCM = 2 (8%)	Pneummonia = 1 (4%)	not specified = 3 (12%)
**Clinical manifestations**				
	Weight loss = 72,7%		Expectoration = 50,0%	Cough = 77,3%
	Fever = 27,3%		Dyspnea = 50,0%	Haemoptysis = 63,6%
	Chest pain = 22,7%			
**Planigraphy and chest CT**	BF = 20 (80%) F = 4 (16%) C = 1 (4%)			
**Cytopathological sputum**	4/14 = 28,6%			
**Cytopathological LBA**	7/12 = 58,3%			
**DID admission**	13/20 = 65%			
**Cirurgical procedures**	BT = 7/ 23 (30,4%)		ESL = 4/23 (17,4%)	
**Antifungal treatment**	ITZ = 17/19 (89,5%)	AMB = 3/19 (15,8%)		VCZ = 1/19 (5,3%)

TB: tuberculosis; PCM: paracoccidioidomycosis; CT: computed tomography; DID: double agar gel immunodiffusion test; TB: transbronchial biopsy; RSL: resection segment or lobe; ITZ: itraconazole; AMB: amphotericin B; VCZ: voriconazole.

### Determination of the *cut-off* point

**Fig 1:**
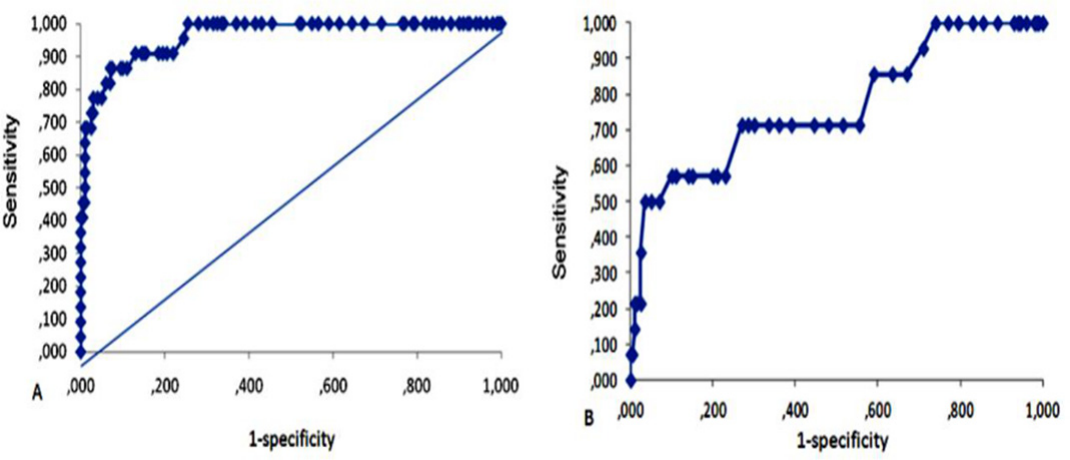
Receiver operator characteristic curve obtained for determining the *cut-off* point of the serum level of anti-*Aspergillus* antibodies, using *A*. *fumigatus* antigen (A) and *A*. *fumigatus*, *A*. *flavus* and *A*. *niger* antigen pool (B), based on 22 patients with chronic pulmonary aspergillosis and 200 healthy blood donors from the same region.

The *cut-off* points were an OD of 0.120 and 0.130 when the *A*. *fumigatus* antigen (A) was used and an OD of 0.090 and 0.100 with the *Aspergillus* spp. antigen *pool* (B) and confidence intervals of 95% and 99%, respectively (Fig 1).

### Determination of the serum dilution to be used

The serum dilution was chosen by comparing the *cut-off* values obtained using sera diluted to 1/25, 1/50 and 1/100. The *cut-off* values, presented as the mean and standard deviation, were as follows: a) 1/25 dilution: 0.130 ± 0.0237; b) 1/50 dilution: 0.113 ± 0.0215; c) 1/100 dilution: 0.121 ± 0.0313. These values did not differ, so the *A*. *fumigatus* antigen (p = 0.26) was used. The *cut-off* values with the *A*. *fumigatus*, *A*. *flavus* and *A*. *niger* antigen pool were as follows: a) 1/25 dilution: 0.0796 ± 0.0118; b) 1/50 dilution: 0.0874 ± 0.0239; c) 1/100 dilution: 0.0854 ± 0.0159. These values did not differ (p = 0.49), so the serum diluted at 1/100 was chosen because the volume spent in the reactions would be lower.

### Accuracy parameters

The use of an antigen *pool* led to a trend of increasing DID sensitivity of 14%, while the other accuracy parameters remained unchanged (Table 2). A positive DID test increased by 90.9 to 118.2 times the likelihood of the pre-test diagnosis being aspergillosis, depending on the antigen used (Table 2). The DID sensitivity in patients with aspergilloma (76.5%) was greater than that of patients with CPA, assessed as a whole.

**Table 2:** Accuracy parameters of immunodiffusion and enzyme-linked immunosorbent assay tests.

Serological tests	S (%)	E (%)	PPV (%)	NPV (%)	PLR (CPRL)	NLR
DID 1	45,5	100,0	100,0	93,3	90,9	0,5
DID 2	59,1	100,0	100,0	95,7	118,2	0,4
ELISA 1 (0,120)	81,8	94,0	60,0	97,9	13,6	0,2
ELISA 1 (0,130)	72,7	97,0	76,2	97,0	29,1	0,3
ELISA 2 (0,090)	86,4	96,5	73,1	98,5	24,7	0,1
ELISA 2 (0,100)	59,1	99,5	92,9	95,7	118,2	0,4

DID:double agar gel immunodiffusion; ELISA: enzyme-linked immunosorbent assay; 1: antigen the *Aspergillus fumigatus*; 2: *pool* de antigens the *A*. *fumigatus*, *A*. *flavus* e *A*. *niger*; 0,120, 0,130, 0,090 e 0,100 –*cut-off* values; S: sensitivity, E: specificity; PPV / NPV: positive and negative predictive values; PLR: positive likelihood ratio; CPRL: corrected positive likelihood ratio; NLR: negative likelihood ratio. Subjects: 22 patients with chronic aspergillosis and 200 healthy controls.

In the ELISA, using a higher *cut-off* point led to decreased sensitivity for both types of antigens but also to increased PPV and PLR (Table 2). In addition, the use of the antigen pool also led to increased PPV and PLR but decreased sensitivity when the *cut-off* was higher (Table 2). The ELISA showed higher sensitivity than DID. Regardless of the antigen preparation used, a positive DID test increased much more the chance of pre-test diagnosis than did a positive ELISA test, as indicated by the PLRs (Table 2). The indices, determined as the ratio between the optical density observed in every sample and the *cut-off*, were 0.173 and 0.420 in the negative samples and ranged from 0.63 to 5.72 for the positive ones (median = 2.08). Finally, using the higher *cut-off*, there was decreased sensitivity, which approached the one presented by DID, but with a slight increase in specificity.

### Cross-reactivity

The detection of anti-*Aspergillus* antibodies by DID test in sera from patients with other granulomatous diseases was negative in almost all of them (Table 3). The ELISA test showed a greater prevalence of cross-reactivity, which was lower when the highest *cut-off* point was used. Cross-reactivity was more frequent in sera from patients with PCM and almost absent in those with CRC (Table 3).

**Table 3:** Prevalence (percentage) of cross-reactions observed in immunodiffusion and enzyme-linked immunosorbent assay tests.

Disease	Patient number	DID 1	DID 2	ELISA 1 (0,120)	ELISA 1 (0,130)	ELISA 2 (0,090)	ELISA 2 (0,100)
**TBC**	28	0,0	0,0	21,4	10,7	10,7	0,0
** **				[0,121–0,128]	[0,138–0,156]	[0,093–0,099]	
**HST**	23	0,0	8,7	30,4	8,7	21,7	13,0
** **			[undiluted—1/4]	[0,120–0,128]	[0,132–0,138]	[0,091–0,094]	[0,103–0,115]
**PCM**	50	2,0	0,0	40,0	36,0	62,0	52,0
** **		[undiluted]		[0,120–0,128]	[0,131–0,181]	[0,091–0,097]	[0,101–0,140]
**CRC**	20	0,0	0,0	0,0	0,0	20,0	0,0
** **						[0,091–0,098]	

DID: double agar gel immunodiffusion test; ELISA: enzyme-linked immunosorbent assay; 1: antigen of *Aspergillus fumigatus*; 2: *pool* of antigens: *A*. *fumigatus*, *A*. *flavus* e *A*. *niger*, () *cut-off* the test; TBC: tuberculosis; HST: histoplasmosis; PCM: paracoccidioidomycosis; CRC: cryptococcosis; [] range.

### Comparison of the sensitivity of the tests

The comparison of the sensitivity of the different diagnostic tests indicated higher frequencies for ELISA using both types of antigen and the lowest frequency for DID_1_ with *A*. *fumigatus* antigen. GM and DID_2_ showed intermediate frequency, which did not differ from the others (Table 4).

**Table 4:** Qualitative results of serological tests performed in 16 patients with chronic aspergillosis. Comparisons carried out among the double agar gel immunodiffusion test (DID) and the enzyme-linked immunosorbent assay (ELISA) using two antigenic preparations, and galactomannan (GM). Comparisons performed by Cochran Q test, McNemar test, and the bionomial test.

Number of order	Patient number	DID_1_	DID_2_	ELISA_1_	ELISA_2_	GM
**1**	**2**	-	-	+	+	+
**2**	**3**	+	+	+	+	-
**3**	**4**	+	+	+	+	+
**4**	**5**	+	+	+	+	+
**5**	**6**	+	+	+	+	+
**6**	**7**	-	-	-	-	+
**7**	**8**	-	-	-	-	+
**8**	**10**	+	+	-	+	+
**9**	**11**	-	+	+	+	-
**10**	**12**	-	+	+	+	+
**11**	**13**	-	-	+	+	+
**12**	**14**	+	-	+	+	+
**13**	**16**	+	-	+	+	+
**14**	**17**	+	+	+	+	+
**15**	**18**	-	-	+	+	+
**16**	**19**	+	+	+	+	+
**Sensitivity (%)**		**56.3** ^**b**^	**56.3** ^**b**^	**81.3** ^**ab**^	**87.5** ^**a**^	**87.5** ^**a**^

- no reagent; +: reagent.

*1-Aspergillus fumigatus* antigen

*2-Pool* of antigens from *Aspergillus fumigatus*, *Aspergillus niger* and *Aspergillus flavus*

*Q = 119*.*34* (*p*<0.00001)

DID_1_ *vs* DID_2_: p = 0.36; DID_1_ *vs* ELISA_1_: p = 0.11; DID_1_ *vs* ELISA_2_: p = 0.03; DID_1_ *vs* GM: p = 0.06

DID_2_ *vs* ELISA_1_: p = 0.11; DID_2_ *vs* ELISA_2_: p = 0.03; DID_2_ *vs* GM: p = 0.09; ELISA_1_ *vs* ELISA_2_: p = 0.50; ELISA_1_ *vs* GM: p = 0.50; ELISA_2_ *vs* GM: p = 0.36

Frequencies followed by the same letter do not differ (*p*>0.05); frequencies followed by different letters are statistically different (*p*≤0.05) presented a tendency a statistical difference (0.05 < *p* ≤ 0.10).

The agreement between the positivity of the GM test and the detection of antibodies was always small, regardless of the method and the antigen used. The strongest agreement was observed between the ELISA_1_ and ELISA_2_. The agreement between the DID tests with different antigens and DID with ELISA was appreciable overall (Table 5).

**Table 5:** Degree of agreement of diagnosis tests in 22 patients with chonic aspergillosis. Comparison 2x2 using the kappa test.

Paired tests (A *vs* B)	Patients (number)					Kappa statistic		
	Total	A+B+	A-B-	A+B-	A-B+	Value	Confidence interval 95%	Strength of agreement
**DID**_**1**_ ***vs* DID** _**2**_	22	8	7	2	5	0.37	[0.00–0.76]	fair
**DID**_**1**_ ***vs* ELISA** _**1**_	22	9	3	1	9	0.14	[0.00–0.53]	slight
**DID**_**1**_ ***vs* ELISA** _**2**_	22	10	3	0	9	0.23	[0.00–0.62]	fair
**DID**_**1**_ ***vs* GM**	16	8	1	1	6	0.03	[0.00–0.57]	slight
**DID**_**2**_ ***vs* ELISA** _**1**_	22	12	3	1	6	0.28	[0.00–0.72]	fair
**DID**_**2**_ ***vs* ELISA** _**2**_	22	12	2	1	7	0.16	[0.00–0,63]	slight
**DID**_**2**_ ***vs* GM**	16	7	0	2	7	0.00	[0.00–0.30]	slight
**ELISA**_**1**_ ***vs* ELISA** _**2**_	22	17	2	1	2	0.49	[0.00–1.00]	moderate
**ELISA**_**1**_ ***vs* GM**	16	11	0	2	3	0.00	[0.00–0.68]	slight
**ELISA**_**2**_ ***vs* GM**	16	12	0	2	2	0.00	[0.00–0.83]	slight

DID: Double agar gel immunodiffusion test; ELISA: enzyme-linked immunosorbent assay; GM: Galactomannan

1*-Aspergillus fumigatus* antigen

2*-Pool* of antigens from *Aspergillus fumigatus*, *Aspergillus niger* and *Aspergillus flavus*

### Evaluation of the serological follow-up of patients under treatment with itraconazole

**Fig 2:**
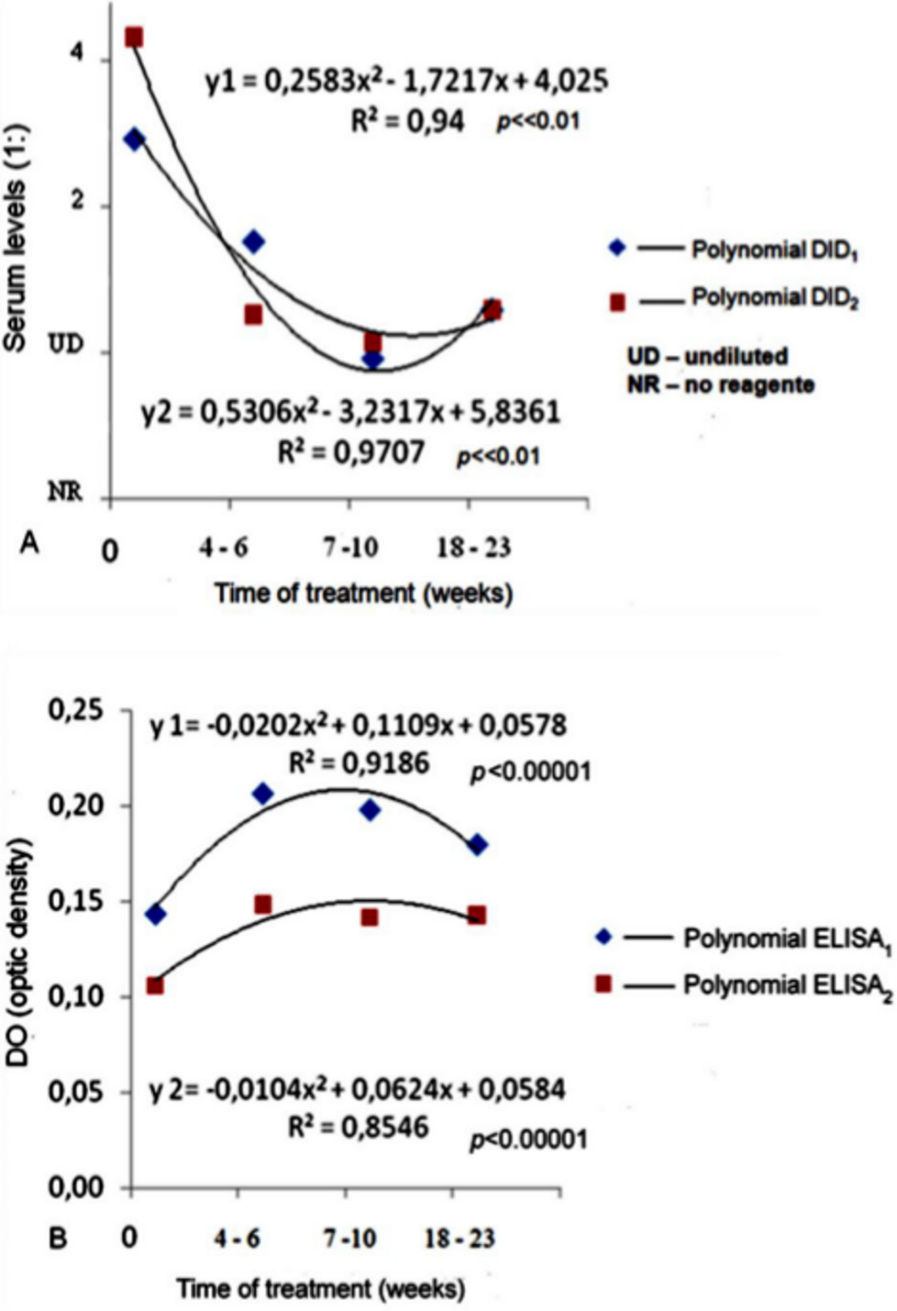
Regression analysis representing changes in serum levels of anti-*Aspergillus* antibodies as a function of the antifungal treatment period in 10 patients with chronic pulmonary aspergillosis. (A) Curve representing the progression of serum levels determined by DID test with the *A*. *fumigatus* antigen and *Aspergillus* spp. antigen pool. (B) Curve representing the progression of serum levels determined by ELISA using *A*. *fumigatus* antigen and *Aspergillus* spp. antigen *pool*.

The regression curves obtained in the follow-up of patients with positive initial serology according to DID_1_ and DID_2_ exhibited the same pattern as a function of treatment time, with decreased serum levels (*p*<0.01). The curves obtained from ELISA were different from those observed with DID because they showed a slight increase in serum antibody levels, more evident when the *A*. *fumigatus* antigen was used (*p*<0.00001), as shown in Fig 2.

The characterization of the 10 patients evaluated and treated showed clinical cure or improvement in all cases, but with persistence of aspergilloma in three patients (Table 6). The mortality rate was 30%; two patients showed persistence of aspergilloma and the third, fibrotic scars (Table 6).

**Table 6:** Progress of 10 patients after treatment, as to age and clinical, roentnologic, and global outcome.

Case number	Age (years)	Treatment lenght (months)	Clinical outcome	Roentnologic outcome	Global outcome
1	51	9	Clinical improvement	Persistence of aspergilloma	Died
2	57	61	Clinical cure	Persistence of aspergilloma	Alive
3	55	86	Clinical cure	Aspergilloma disappeared	Alive
4	43	12	Clinical cure	Fibrotic scars	Alive
5	74	49	Clinical cure	Aspergilloma disappeared	Alive
6	67	17	Clinical cure	Aspergilloma disappeared	Alive
7	44	12	Clinical cure	Persistence of aspergilloma	Alive
8	80	2	Clinical improvement	Persistence of aspergilloma	Died
9	33	3,5	Clinical cure	Aspergilloma disappeared	Alive
10	76	8,5	Clinical improvement	Fibrotic scars	Died

### Clinical progress. Comparison with double agar gel immunodiffusion test

**Fig 3:**
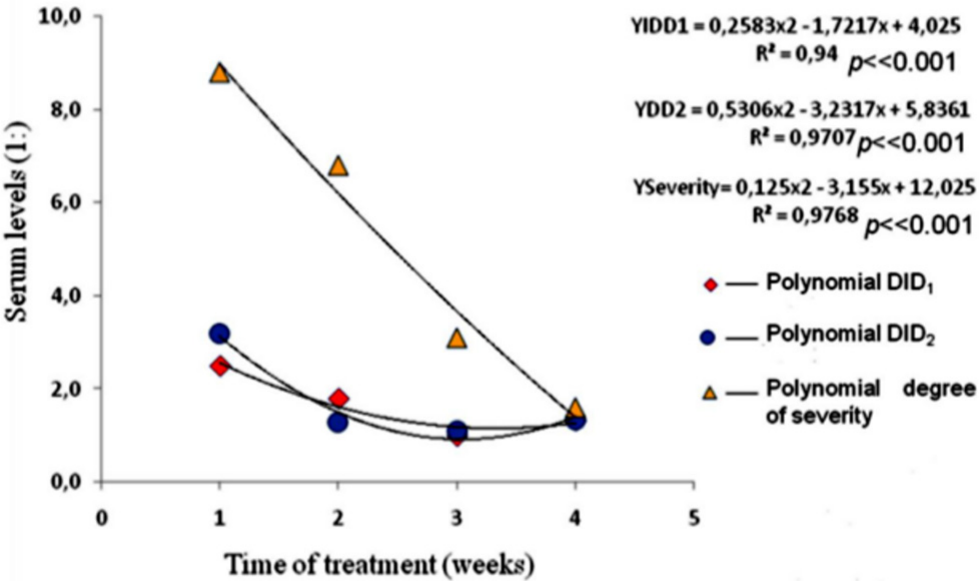
Regression analyses showing the decreasing antibody serum levels anti-*Aspergillus*, and evident clinical improvement after introduction of antifungal treatment. The regression curves are different.

Clinical follow-up, evaluated by using complaints scores, showed evident clinical improvement after introduction of antifungal treatment, as it was observed with the decreasing antibodies serum levels, determined by DID test. However, the clinical curve was different from the DID test ones, taken together (Fig 3).

## Discussion

CPA has several clinical manifestations: aspergilloma, also called aspergilloma, CCPA and CFPA. The pulmonary sequelae caused by TB are an important antecedent of CPA [3, 33]. Worldwide, more than 36 million people have been cured of TB from 1995 to 2008, and 9 million new cases per year have been diagnosed. The British Thoracic and Tuberculosis Association noted that 6% of patients with open-scar tuberculous cavities developed aspergillomas in three years, with a mortality of 6% per year [34–36]. In another study, the mortality rate was 31% in 5 years and 56% in 10 years; of the 27 patients who died, 3 of them died due to hemoptysis, 7 due to surgical complications, 6 due to chronic respiratory failure, 6 due to acute pneumonia and 5 due to chronic suppurative pneumonia [34]. In Brazil, in 2003, the incidence of TB was of 44.4 new cases/100,000 inhabitants. In 2013, 71,123 new cases were reported, with an incidence of 35.4 cases/100,000 inhabitants [35]. All these data indicate the importance of CPA for public health and the high number of patients with underlying diseases that favor its appearance. These data also suggest that the prevalence of CPA in our environment must be much higher than the number of cases referred to university hospitals and therefore that many of them have not been diagnosed.

However, it should be noted that simple identification in the sputum of fungi that colonize the bronchial tree is not confirmation of the etiologic diagnosis of lung injury, which occurs with *Cryptococcus* and *Aspergillus* spp. The solution to this problem depends on surgical procedures, such as lung biopsy or resection of lung injury and serological tests. Surgical procedures are often aggressive and sometimes contraindicated. Serological tests can be performed using several methods and different antigen preparations. In addition, the presence of GM and IgG in the serum of these patients can be evaluated.

Thus, the present study aimed to compare the accuracy parameters of two serological tests (DID and ELISA), using two antigen preparations (*A*. *fumigatus* antigen and *A*. *fumigatus*, *A*. *flavus* and *A*. *niger* antigen pool) and qualitative detection of serum GM in patients with CPA. Moreover, we evaluated the serological progression of the patients after introduction of the antifungal treatment.

Several serological methods, such as complement fixation reactions, gel precipitation reactions, latex particle agglutination, electrophoretic tests and enzymatic immunoassays have been evaluated for the diagnosis of aspergillosis [10, 37]. The immunodiffusion reaction in agar gel is the most used method due to the simplicity of execution, reproducibility [38], low prevalence of cross reactions and a high positive likelihood ratio, which are the reasons that led to its standardization for the detection of anti-*Aspergillus* antibodies in most clinical laboratories. However, the low sensitivity and the long period necessary to the reading of the slides are drawbacks of this method. However, the antigen concentration required to detect precipitins in the DID is a critical factor of this reaction [39], which can only be read 96 hours after the slides are prepared. With the advent of enzyme immunoassays, the introduction of ELISA test has been encouraged in routine laboratories, given its sensitivity, speed of execution and direct measurement of antibody levels [26, 37–40].

In the present study, the prevalence of patients with aspergilloma among those with CPA, the males, and those with cough, weight loss and hemoptysis as clinical manifestations of pulmonary TB as pre-existing disease confirms the findings of other authors [20, 34–36]. The use of serum diluted to 1/100 aimed to better use the serum of each patient, given that the results did not differ from those observed at 1/25 and 1/50 dilutions. The sensitivity of DID test observed in patients with aspergilloma in the present study (76.5%) was lower than that found by other authors (91.0 to 98.0%) [10, 41–43]. The finding of high specificity and PPV confirms those same studies [10, 41–43]. The PLR or verisimilitude ratio, which was not evaluated by other authors, was very high, which makes the post-test likelihood almost a certainty. This fact justifies defining a confirmed case in patients who have positive DID test with suggestive clinical and radiological manifestations.

The sensitivity of ELISA test was higher than that of DID test, although without reaching 95%, confirming the findings of other authors [10, 41–43]. The higher sensitivity of ELISA test can be explained by the identification of antibodies that do not precipitate on agar or agarose gel but are immobilized on polystyrene plates [37]. The PLR was high, although lower than that observed with DID test, thus strengthening the CPA pre-test hypothesis.

The antigen preparation used is essential to evaluate the accuracy of serological tests [10]. Because different preparations were used in different studies, comparisons cannot be direct. Nevertheless, the overall set of results can be considered harmonious. The use of an *Aspergillus* spp. antigen pool is well indicated because cases of aspergillosis by *A*. *fumigatus*, *A*. *flavus*, *A*. *niger*, *A*. *nidulans* and *A*. *terreus* have been described, though with a predominance of the first [10, 41–42,44–45].

The cross-reactivity measured in sera from patients with PCM, HST, TB and CRC were infrequent with DID test, present only with HST and PCM. In contrast, ELISA test showed false-positive results with HST, TB and PCM, and ELISA_2_ test showed false positives with the four granulomatous diseases. These findings can be explained by the production of GM by *Paracoccidioides brasiliensis*, *Histoplasma capsulatum* and *Cryptococcus neoformans* [46–52]. Cross-reactivity with sera from patients with active TB must be carefully evaluated because TB is often the underlying disease of CPA and anti-*Mycobacterium tuberculosis* antibodies may persist for a long time.

The serum GM detection showed the same sensitivity as ELISA test. Considering that immunodiffusion tests present high specificity but low sensitivity, and the ELISA tests present higher sensitivity but an elevated percentage of cross-reactions with paracoccidioidomycosis and tuberculosis. Considering that tuberculosis is usually an underlying disease for chronic pulmonary aspergillosis, we decided to include galactomannan in this study. The results were very good, suggesting the determination of galactomannan in new studies. However, this finding must be carefully interpreted due to the possibility of cross-reactivity with other fungal diseases that also affect the lungs [46–52]. Interestingly, *Aspergillus* spp. produce GM, a complex polysaccharide composed of D-galactose and D-mannose, and *C*. *neoformans* also contains a capsular polysaccharide complex, composed of glucuronoxylomannan (GXM) and GMs. GXM is found in body fluids, and its identification allows the diagnosis of CRC [53]. Despite the apparent similarity between these polysaccharides, the cross-reactivity in sera from patients with CRC was much less frequent than in patients with HST or PCM.

The progression of the serum antibody levels evaluated by DID test was different from that of ELISA test, although both produced polynomial curves. While the curve shown in the DID evaluation was descending and with no difference depending on the antigen preparation, the curve produced by ELISA test was slightly ascending and behaved differently according to the antigen used. Considering that (a) DID test measures only precipitating antibodies in agar/agarose gel and ELISA test identifies non-precipitating antibodies that are immobilized on polystyrene plates [37] and that (b) patients with aspergilloma have increased serum IgG as determined by ELISA test [26], which continue to increase after the introduction of the antifungal agent, the behavior of the two serological progression curves after introduction of the treatment can be explained. This hypothesis can be tested by assessing the progression of the serological curve of anti-*Aspergillus* IgG, as currently there is a kit available for this determination, which will be performed soon. The decrease in serum antibody levels, determined by DID test, after the introduction of an effective treatment, was also observed by other authors [5, 43, 54, 55]. Thus, based on the results available, the follow-up of patients must be performed using DID.

The present study has some limitations: a small sample, as a result of the small number of referred patients with the CPA hypothesis; the *Aspergillus* species was rarely identified; and, finally, the anti-*Aspergillus* IgG was not measured in the serum of these patients.

## References

[1] Denning DW. Invasive aspergillosis. Clin Infect Dis. 1998;26: 781–803. 9564455 10.1086/513943

[2] Zmeili OS, Soubani AO. Pulmonary aspergillosis: a clinical update. QJM. 2007;100: 317–334. 17525130 10.1093/qjmed/hcm035

[3] Denning DW, Pleuvry A, Cole DC. Global burden of chronic pulmonary aspergillosis as a sequel to pulmonary tuberculosis. Bull World Health Organ. 2011;89: 864–872. 10.2471/BLT.11.089441 22271943 PMC3260898

[4] Smith NL, Denning DW. Underlying conditions in chronic pulmonary aspergillosis including simple aspergilloma. Eur Resp J. 2001;37: 865–872. 10.1183/09031936.00054810 20595150

[5] Walter JE, Jones RD. Serologic test in diagosis of aspergillosis. Dis Chest. 1968;53: 729–735. 5653748 10.1378/chest.53.6.729

[6] Denning DW, Follansbee SE, Scolaro M, Norris S, Edelstein H, Stevens DA. Pulmonary aspergillosis in the acquired immunodeficiency syndrome. N Engl J Med. 1991;324: 654–662. 1994248 10.1056/NEJM199103073241003

[7] Kawamura S, Shigefumi M, Kazunori T, Takayoshi T, Shigeru K. Clinical evaluation of 61 patients with pulmonary aspergilloma. Intern Med. 2000;39: 209–212. 10772121 10.2169/internalmedicine.39.209

[8] Restrepo A. La prueba de inmunodiffusion en el diagnostic de la paracoccidioidomicosis. Sabouraudia. 1966;4: 223–230. 4959861

[9] Coleman RM, Kaufaman L. Use of the immunodiffusion test in the serodiagnosis of Aspergillosis. Appl Microbiol. 1972;23: 301–308. 4622826 10.1128/am.23.2.301-308.1972 PMC380335

[10] Stynen D, Goris A, Sarfati J, Latgé JP. A new sensitive sandwich enzyme-linked immunosorbent assay to detect galactofuran in pacients with invasive aspergillosis. J Clin Microbiol. 1995;33: 497–500. 7714217 10.1128/jcm.33.2.497-500.1995 PMC227977

[11] Swanink CMA, Meis JFG, Rijs AJMM, Donnelly JP, Verweij PE. Specificity of a Sandwich enzyme-linked immunosorbent assay for detecting *Aspergillus* galactomannan. J Clin Microbiol. 1997;35: 257–260. 8968919 10.1128/jcm.35.1.257-260.1997 PMC229550

[12] Aquino VR, Goldani LZ, Pasqualotto AC. Update on the contribution of galactomannan for the diagnosis of invasive aspergillosis. Mycopathologia. 2007;163: 191–202. 17410480 10.1007/s11046-007-9010-2

[13] Walsh TJ, Anaissie EJ, Denning DW, Herbrecht R, Kontoyiannis DP, Marr KA, et al Treatment of aspergillosis: clinical practice guidelines of the infectious diseases society of america. Clin Infect Dis. 2008;46: 327–360. 10.1086/525258 18177225

[14] Kilman JW, Ahn C, Andrews NC, Klassen K. Surgery for pulmonary aspergillosis. J Thorac Cardiovasc Surg. 1969;57: 642–647. 5782409

[15] Daly RC, Pairolero PC, Piehler JM, Trastek VF, Payne WS, Bernatz PE. Pulmonary aspergilloma: results of surgical treatment. J Thorac Cardiovasc Surg. 1986;92: 981–988. 3097424

[16] Jewkes J, Kay PH, Paneth M, Citron KM. Pulmonary aspergilloma: analysis of prognosis in relation to haemoptysis and survey of treatment. Thorax. 1983;38: 572–578. 6612647 10.1136/thx.38.8.572 PMC459613

[17] Massard G, Roeslin N, Wihlm JM, Dumont P, Witz JP, Dumond P, et al Pleuropulmonary aspergilloma: clinical spectrum and results of surgical treatment. Ann Thorac Surg. 1992;54: 1159–1164. 1449303 10.1016/0003-4975(92)90086-j

[18] Tsubura E. Multicenter clinical trial of itraconazole in the treatment of pulmonary aspergilloma. Pulmonary aspergilloma study group [abstract]. Kekkaku. 1997;72: 557–564. 9386354

[19] Restrepo A, Múnera AI, Arteaga ID, Gómez I, Tabares AM, Patiño MM. Itraconazole in the treatment of pulmonary aspergilloma and chonic pulmonary aspergllosis In: Bossche HV, Mackenzie D, Cauwenberg G. *Aspergillus* and Aspergillosis. New York: Plenum Press; 1988 pp. 253–265.

[20] Denning DW, Tucker RM, Hanson LH, Stevens DA. Treatment of invasive aspergillosis with itraconazole. Am J Med. 1989;86: 791–800. 2543220 10.1016/0002-9343(89)90475-0

[21] Denning DW, Riniotis K, Dobrashian R, Sambatakou H. Chronic cavitary and fibrosing pulmonary and pleural aspergillosis: case series, proposed nomenclature change, and review. Clin Infect Dis. 2003;37 Suppl 3: S265–280. 12975754 10.1086/376526

[22] Camuset J, Nunes H, Dombret MC, Bergeron A, Henno P, Philippe B, et al Treatment of chronic pulmonary aspergillosis by voriconazole in non immunocompromised patients. Chest. 2007;131: 1435–1441. 17400661 10.1378/chest.06-2441

[23] Pedroso RS, Candido RC. Diagnóstico laboratorial da criptococose. NewsLab. 2006;77: 94–102.

[24] De Beule K, De Doncker P, Cauwenbergh G, Koster M, Lengendre R, Blatchford N, et al The treatment of aspergillosis and aspergilloma with itraconazole, clinical results of na open international study (1982–1987). Mycosis. 1988;31: 476–485. 10.1111/j.1439-0507.1988.tb03653.x 2849056

[25] Sepulveda R, Longboottom JL, Pepys J. Enzyme-linked immunosorbent assay (ELISA) for IgG and IgE antibodies to protein and polysaccharide antigens of *Aspergillus fumigatus* . Clin Allergy. 1979;9: 359–371. 113131 10.1111/j.1365-2222.1979.tb02494.x

[26] Mantyjärvi RA, Jousilahti P, Katila ML. Antibodies to *Aspergillus fumigatus* in farmer’s lung patients measured by enzyme-linked immunosorbent assay. Clin Allergy. 1980;10: 187–194. 6993041 10.1111/j.1365-2222.1980.tb02096.x

[27] Fletcher RH, Fletcher SW. Diagnóstico In: Epidemiologia clínica: elementos essenciais. Porto Alegre: Artes Médicas; 2006 pp. 56–81.

[28] Frey A, Canzio JD, Zurakiwski D. A statistically defined endpoint titer determination method for immunoassays. J Immunol Methods. 1998;221: 35–41. 9894896 10.1016/s0022-1759(98)00170-7

[29] Maia Filho HS, Cunha AJLA. Diagnóstico In: Gomes MM. Medicina baseada em evidências: princípios e práticas. Rio de Janeiro: Editora Reichmann & Affonso; 2001 pp. 81–94.

[30] Curi PR. Provas não-paramétricas para mais de duas amostras In: Metodologia e análise da pesquisa em ciências biológicas. Botucatu: Tipomic; 1997 pp. 220–228.

[31] Siegel S. A prova de McNemar para a significância das mudanças In: Estatística não-paramétrica para as ciências do comportamento. São Paulo: McGraw-Hill do Brasil; 1975 pp. 69–74.

[32] Landis JR, Koch GG. The measurement of observer agreement for categorical data. Biometrics. 1977;33: 159–174. 843571

[33] Hossain A, Islam QT, Siddiqui MR, Tamanna N, Sina H, Rahman YU, et al Pulmonary aspergilloma. J Med. 2009;10: 149–151.

[34] Jewkes J, Kay PH, Paneth M, Citron K. Pulmonary aspergilloma: analysis of prognosis in relation to haemoptysis and survey of treatment. Thorax. 1983;38: 572–578. 6612647 10.1136/thx.38.8.572 PMC459613

[35] Ministério da Saúde. Secretaria de Vigilância em Saúde. O controle da tuberculose no Brasil: avanços, inovações e desafios. Bol Epidemiol. 2014;45: 2–14. Available: http://portalsaude.saude.gov.br/images/pdf/2014/maio/29/BE-2014-45—2—tb.pdf

[36] Aspergilloma and residual tuberculosis cavities–the results of a resurvey: a report from the research committee of the British Thoracic and Tuberculosis Association. Tubercle. 1970;51: 227–245. 5495645

[37] Kauffman HF, Beaumont FB, Meurs H, van der Heide S, de Vries K. Comparison of antibody measurments against *Aspergillus fumigatus* by means of double-diffusion and enzyme-linked immunosirbent assay (ELISA). J Allergy Clin Immunol. 1983;72: 255–261. 6411795 10.1016/0091-6749(83)90029-5

[38] Froudist JH, Harnett GB, McAller R. Comparison of immunodifusion and enzyme linked immunosorbent assay for antibodies to four *Aspergillus* species. J Clin Pathol. 1989;42: 1215–1221. 2511230 10.1136/jcp.42.11.1215 PMC501985

[39] Richardson MD, Stubbins JM, Warnock DW. Rapid enzyme-linked immunosorbent assay (ELISA) for *Aspergillus fumigatus* antibodies. J Clin Pathol. 1982;35: 1134–1137. 6813358 10.1136/jcp.35.10.1134 PMC497897

[40] Shale DJ, Faux JA. The evaluation of a quantitative enzyme-linked immunosorbent assay (ELISA) for anti-*Aspergillus fumigatus* IgG. J Immunol Methods. 1985;7: 197–205. 10.1016/0022-1759(85)90032-8 3884714

[41] Campbell MJ, Clayton YM. Bronchopulmonary aspergillosis. A correlation of the clinical and laboratory findings in 272 patients investigated for bronchopulmonary aspergillosis. Am Rev Resper Dis. 1964;89: 186–196. 10.1164/arrd.1964.89.2.186 14120084

[42] Longbottom JL, Pepys J. Pulmonary aspergillosis: diagnostic and immunological significance of antigens and C-substance in *Aspergillus fumigatus* . J Pathol Bacteriol. 1964;88: 141–151. 14194971 10.1002/path.1700880119

[43] Stallybrass FC. The precipitin test in human aspergillosis. Mycopathol Appl. 1963;21: 272–278. 10.1007/BF02052580 14111106

[44] Longbottom JL, Pepys J, Cline FT. Diagnostic precipitin test in aspergillus pulmonary mycetoma. Lancet. 1964;1: 588–589. 14104489 10.1016/s0140-6736(64)91335-2

[45] Ferreira-da-Cruz MF, Wanke B, Pirmez C, Galvão-Castro B. *Aspergillus fumigatu*s fungus ball in hospitalized patients with chonic pulmonary disease. Usefulness of double immunodiffusion test as a screening procedure. Mem Inst Oswaldo Cruz. 1988;83: 357–360. 3152275 10.1590/s0074-02761988000300013

[46] Xavier MO, Pasqualotto AC, Cardoso IC, Severo LC. Cross-reactivity of *Paracoccidioides brasiliensis*, *Histoplasma capsulatum*, and *Cryptococcus* species em the commercial Platelia *Aspergillus* enzyme immunoassay. Clin Vaccine Immunol. 2009;16: 132–133. 10.1128/CVI.00310-08 19020109 PMC2620656

[47] Aquino VR, Goldani LZ, Pasqualotto AC. Update on the contribution of galactomannan for diagnosis of invasive aspergillosis. Mycopathologia. 2007;163: 191–202. 17410480 10.1007/s11046-007-9010-2

[48] Dalle F, Charles PE, Blanc K, Caillot D, Chavanet P; Dromer F, et al *Cryprococcus neoformans* galactoxylomannan contains an epitope(s) that is cross-reactive with *Aspergillus* galactomannan. J Clin Microbiol. 2005;43: 2929–2931. 15956422 10.1128/JCM.43.6.2929-2931.2005 PMC1151935

[49] Giacchino M, Chiapello N, Bezzio S, Fagioli F, Saracco P, Alfarano A, et al *Aspergillus* galactomannan enzyme-linked immunosorbent assay cross-reactivity caused by invasive *Geotrichum capitatum* . J Clin Microbiol. 2006;44: 3432–3434. 16954294 10.1128/JCM.00856-06 PMC1594704

[50] Huang Y, Hung C, Liao C, Sun H, Chang S, Chen Y. Detection of circulating galactomannan in serum samples for diagnosis of *Penicillium marneffei* infection and cryptococcosis among patients infected with human immunodeficiency virus. J Clin Microbiol. 2007;45: 2858–2862. 17596363 10.1128/JCM.00050-07 PMC2045252

[51] Narreddy S. False-positive *Aspergillus* galactomannan (GM) assay in histoplasmosis. J Infect. 2008;56: 80–81. 17983658 10.1016/j.jinf.2007.09.013

[52] Wheat JL, Hackett E, Durkin M, Connolly P, Petraitiene R, Walsh TJ, et al Histoplasmosis-associated cross-reactivity in the BioRad Platelia *Aspergillus* enzyme immunoassay. Clin Vaccine Immunol. 2007;14: 638–640. 17344352 10.1128/CVI.00479-06 PMC1865624

[53] De Jesus M, Hackett E, Durkin M, Connolly P, Casadevall A, Petraitiene R, et al Galactoxylomannan does not exhibit cross-reactivity in the Platelia *Aspergillus* enzyme immunoassay. Clin Vaccine Immunol. 2007;14: 624–627. 17360857 10.1128/CVI.00368-06 PMC1865626

[54] Halweg H, Ciszek J, Krakwka P. The reversal of serological reactions in patients with pulmonary and pleural aspergillosis after treatment. Tubercle. 1968;49: 404–409. 4975545 10.1016/s0041-3879(68)80021-2

[55] Slavin RG, Million L, Cherry J. Allergic bronchopulmonary aspergillosis: characterization of antibodies and results of treatment. J Allergy. 1970;46: 150–155. 4990018 10.1016/0021-8707(70)90093-6

